# Toll-like-Receptor Gene Polymorphisms in Tunisian Endemic Pemphigus Foliaceus

**DOI:** 10.1155/2020/6541761

**Published:** 2020-11-05

**Authors:** O. Abida, E. Bahloul, N. Elloumi, A. Toumi, Safa Tahri, M. Ben Jmaa, R. Fakhfakh, N. Mahfoudh, H. Turki, H. Masmoudi

**Affiliations:** ^1^Research Laboratory “Autoimmunity, Cancer and Immunogenetics” (LR18SP12), Immunology Department, Habib Bourguiba University Hospital of Sfax, Tunisia; ^2^Dermatology Department, Hedi Chaker University Hospital of Sfax, Sfax, Tunisia; ^3^Immunology Department, Hedi Chaker University Hospital of Sfax, Sfax, Tunisia

## Abstract

Pemphigus foliaceus (PF) is considered to be caused by the combined effects of susceptibility genes and environmental triggers. The polymorphisms of Toll-like receptors (TLRs) genes have been associated with the risk of various autoimmune diseases. The aim of this study was to evaluate the potential association of *TLR2*-*3*-*4* and *7* gene polymorphisms with Tunisian PF. Fourteen polymorphisms were analyzed in 93 Tunisian PF patients compared to 193 matched healthy controls: rs5743703-rs5743709 and (GT)_n_ repeat (*TLR2*); rs5743305, rs3775294, and rs3775291 (*TLR3*), rs4986790 and rs4986791 (*TLR4*); and rs3853839 (*TLR7*). Our results showed that the genetic factors varied depending on the epidemiological feature stratification. In fact, in the whole population, no association with the susceptibility to PF was found. The TLR2 GT repeat seems to be closely associated with PF risk in patients originated from the endemic localities (group 3); the GT_18_ allele and the heterozygous genotype GT_18_/GT_19_ seem to confer risk to endemic PF (*P* = 0.02; OR = 2.3 [1.1-4.9] and *P* = 0.0002, OR = 20 [2.5-171], respectively). In contrast, the GT_23_ repeat could be considered as protector allele (*P* = 0.02, OR = 0.2 [0.06-0.87]). Furthermore, medium GT alleles which induce high promoter activity were also significantly more frequent in patients versus short or long GT repeats (*P* = 0.0018 with OR = 3.26 [1.5-7]). On the other hand, the TLR3-rs574305 AA genotype and A allele were significantly more frequent in patients whose age of the onset was above 35 years (group 2) (*P* = 0.038, OR = 1.78 and *P* = 0.009, OR = 3.92, respectively). Besides, the *TLR4*>rs3775294 A allele was found to be protector only in patients with sporadic features (groups 2 and 4) (*P* = 0.03, OR = 0.57 [0.3-0.9] and *P* = 0.006, OR = 0.24 [0.08-0.74], respectively). No statistically significant difference was observed in the genotypic and allelic frequencies of *TLR-4* and *TLR-7* gene polymorphisms. The present data suggest that *TLR2*and TLR3 polymorphisms are significantly associated with increased susceptibility to PF in the Tunisian population.

## 1. Introduction

The current body of knowledge in the pemphigus foliaceus (PF) field points to an evident multistep model of disease pathogenesis characterized by blister formation and acantholysis which most likely associated with downstream events following the binding of desmoglein-1- (Dsg1-) specific IgG pathogenic autoantibodies (-Abs) [1, 2]. The disease occurs in a genetically susceptible individual who is exposed to a triggering environmental antigen that finally leads, by molecular mimicry, to anti-Dsg1 Abs triggers [3]. Common genetic factors contribute to the different varieties of PF, which share similar immunological characteristics but occur in different environments. However, little is known regarding the environmental trigger. Recent reports provide substantial evidence that the initiation and/or exacerbation of skin lesions could be triggered by microbial organisms [4, 5]. Infectious agents are plausible environmental triggers for autoimmunity in genetically susceptible individuals. Ongoing studies to identify the triggering environmental antigen in PF will be critically important to make significant progress in the disease's therapy [6].

Recent attention has been focused on the Toll-like receptors (TLR) family. TLRs are important innate immune receptors for (i) the identification of invariant and unique conserved molecular patterns among the entire microbial world and (ii) the clearance of invading pathogens [7]. Their expression is not confined to immune cells and has been detected in skin cells such as keratinocytes and melanocytes suggesting that they might be involved in dermatologic disorders [8, 9]. Polymorphic variants of genes implicated in innate immunity such as the TLR family may affect immune responses and hence promote autoaggressive reactions [10]. Current knowledge indicates that genetic risk variants in these receptors alter mRNA expression levels causing disturbance in their function [11]. TLR gene polymorphisms have been studied in the context of various autoimmune diseases in case-control association studies such as rheumatoid arthritis [12, 13] and systemic lupus erythematosus [14, 15].

In fact, mutations that affect the *tlr2* receptor expressions may impair the host response. The most commonly discussed polymorphisms in the *TLR2* (*4q31.3*), the R677W (rs5743704), and the R753Q (rs5743708) have been shown to be associated to several diseases (Tsui et al., 2008; Zhang et al., 2019). The TLR2/G2477A/R753Q has been shown to lead to a decreased cellular activation in the presence of theTLR2 ligand lipopeptide [16]. Furthermore, the presence of a functional intronic polymorphism consisting of guanine-thymine repeats (approximately 100 bp upstream of the translational start site) in the TLR2 gene was reported as a risk factor in rheumatoid arthritis [17]. Indeed, subjects with shorter GT repeats differ from those with longer repeats, and the *TLR2* promotor activity positively correlates with the intron II GT repeat length [13].

On the other hand, variations within the *TLR3* locus (*4q35.1*) are associated with the predisposition to viral infections and to autoimmune diseases such as in systemic lupus erythematosus [14]. Indeed, in the TLR3 gene: rs5743305 (T/A) is located within the promoter region and might affect the transcriptional activity, rs3775291 (C/T) is a nonsynonymous single nucleotide polymorphism (SNP) located within exon 4, and the variant receptor has been shown to be functionally impaired. A study of cell cultures indicated that the TLR3 SNP rs3775296 (in the promoter) and rs3775291 affected the *tlr3*'s cell surface expression and localization and subsequently influenced the NF-*κ*B's cascade induction [18].

In this regard, several studies have examined the associations with *TLR4*+896A/G (Asp299Gly/rs4986790) and +1196C/T (Thr399Ile/rs4986791) polymorphisms and autoimmune disease course like rheumatoid arthritis and chronic psoriasis [12, 19]. Previous findings concluded that these mutations in *TLR4* (*9q33.1*) are associated with the differences in the LPS's responsiveness in humans [20] and demonstrated that the gene sequence changes can alter the ability of the host to respond to environmental stress. TLR4's SNPs might alter disease severity in rheumatoid arthritis by modifying the TLR4 function and/or its gene expression [12].

Furthermore, *TLR7* which is critical to the induction of antiviral immunity is also a key pathogenic factor in systemic lupus erythematosus [21]. The gene encoding *TLR7*mapped in Xp22.2.The rs3853839>G/CSNP of *TLR7* located in the 3′untranslated region of the mRNA conferred the elevated *tlr7* expression at both mRNA and protein levels [15].

Based on these findings and reports, a possible role of TLRs in PF could be suspected, but is yet to be clearly established. Therefore, our study was designed to investigate the association of fourteen TLR genes' polymorphisms (8 in *TLR2*, 3 in *TLR3*, 2 in *TLR4*, and 1 in *TLR7*) with Tunisian endemic PF using a case–control approach.

## 2. Material and Methods

### 2.1. Study Populations

**Figure 1:**
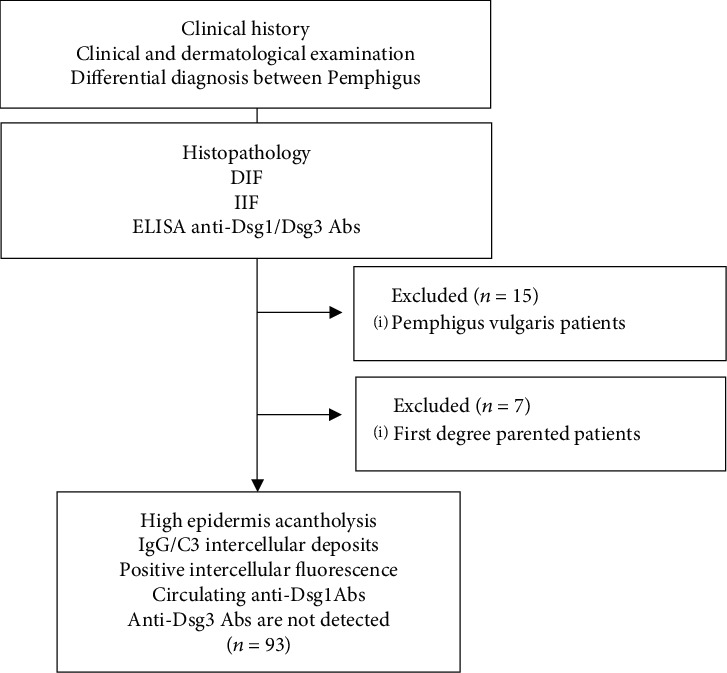
Flowchart for the inclusion and exclusion of the studied patients. DIF: direct immunofluorescence; Dsg: desmoglein, IIF: indirect immunofluorescence.

This genetic retrospective study is a case-control study performed in the Tunisian population since 2003. Patients have been recruited at the Department of Dermatology in the Hedi Chaker University Hospital of Sfax, Tunisia (Figure 1). The diagnosis of PF was confirmed by the clinical presentation, histopathology (acantholysis in the upper epidermis either in the granular layer or immediately below with subcorneal bullous formation), direct immunofluorescence (IgG and C3 deposits most often located on the whole epidermis and less frequently predominant in the upper layers of the epidermis), indirect immunofluorescence (IgG Abs directed against the epithelial cell surface), and ELISA test for circulatory anti-Dsg1 Abs that was positive for all patients [22, 23].

Ninety-three (93) PF patients were matched by age (± 5 years), gender, and geographical origin to one hundred ninety-three (193) healthy controls (HC). Control subjects have no signs of autoimmune or inflammatory disorders. All patients and controls were originated from the southern regions of Tunisia.

The local ethical board of the Habib Bourguiba University Hospital of Sfax approved the study (protocol number of ethical committee, 4/12), and informed consent was obtained from all participants.

### 2.2. Selection of Polymorphisms

To select the most representative genetic variations, tag polymorphisms were selected using the genotyping data from the CEU available from the International Hapmap project and according to their association with the susceptibility to other autoimmune diseases and for their potentially functional relevance. Thus, SNPs could influence the gene expression through changes in the promoter activity, stability of messenger RNA, protein functioning, and/or pathogenic clinical significance. rs5743703/04/05/06/07/08/09 (*TLR2*): rs5743305, rs3775294, and rs3775291 (F412L)(*TLR3*) and rs4986790 (Asp299Gly), rs4986791 (Thr399Ile) (*TLR4*), and rs3853839 (*TLR7*) were selected by these means (Table 1). Additionally, a functional intronic polymorphism consisting of guanine-thymine repeats in *TLR2* was also selected.

### 2.3. TLR Genotyping

Genomic DNA was extracted from whole blood samples using a standard proteinase K digestion and phenol/chloroform extraction procedure. Genotyping was performed using the PCR-RFLP method for all SNPs except the TLR2 exon1 polymorphisms (rs5743703/04/05/06/07 and 09) and the (GT)_n_ microsatellite which were genotyped using the sequencing and the automatic genotyping methods, respectively.

The PCR amplification was carried out in a volume of 25 *μ*l including 1x buffer, 2 mM MgCl2, 0.2–0.4 *μ*mol of each primer (Invitrogen®, CA, USA), 0.12 mM dNTP (Invitrogen®, CA, USA),1 U Taq polymerase (Invitrogen®, CA, USA), and 50 *η*g of DNA template.

Enzymatic digestion was performed in a total of 10 *μ*l mixture reaction containing 1x buffer, 0.1x BSA, and 2 U restriction enzyme (Thermo Fisher®, MA, USA). Primers were designed using primer3 software (http://primer3.ut.ee/). Restriction enzymes were selected using the NEBcutter software (http://nc2.neb.com/NEBcutter2/).

The microsatellite locus was amplified with primer labeled with hexachloro-6-carboxyfluoresceine (HEX) (Table 1) according to published data [24]. The PCR amplification was carried out in a volume of 10 *μ*l including 1x buffer, 2.5 pmol of each primer (Invitrogen®, CA, USA), 10 mM dNTP (Invitrogen®, CA, USA), 1 U AmpliTaq Gold™ DNA Polymerase (Applied Biosystems™, CA, USA), and 1 *η*g of DNA template. Amplified products were run on an ABI prism DNA sequencer (PerkinElmer®, CT, USA), and output file was analyzed using GeneScan softwares analysis.

### 2.4. Statistical Analysis

A case-control analysis was performed using SHESIS software (http://analysis.bio-x.cn) for each SNP and haplotype. Hardy–Weinberg equilibrium (HWE) was assessed in controls using a *χ*2 test with one degree of freedom. A threshold *P* < 0.05 was regarded to indicate deviation from HWE. Odds ratios (OR) and 95% confidence intervals (CI) were calculated for each allele using 2 × 2 contingency tables to estimate the magnitude of association. Fisher's exact test was used for rare allele/genotype. The linkage disequilibrium (LD) coefficients D′ = *D*/*D*_max_ and *r*^2^ values for the pair of the most common alleles at each site were also estimated, and high values of LD were defined as *r*^2^ > 0.33 and *D*′ > 0.7. The significance level of *P* < 0.05 and odds ratios (OR) with 95% confidence intervals (95% CI) was chosen for all sets.

## 3. Results

### 3.1. Study Population

This case-control study enrolled93PF patients with a mean age of 35 years (range, 18–60) and a sex-ratio F/M of 15/1 whose were matched by age (±5 years), gender, and geographical origin to 193HCwith a mean age of 38 years (range, 14–73).

According to our previous data [25], Tunisian endemic PF constitutes a distinct variety of the disease with particular and unique epidemiological characteristics as the disease occurs mainly in young women at a mean age of 35 years. Later, we have showed that this characteristic endemic form occurs in the rural regions of the south and predominantly in the three localities of Moknessy (Sidi Bouzid), Jebenièna (Sfax), and Mereth (Gabès) [26].

It is thus questionable if genetic factors of Tunisian PF could be varied according to the epidemiological feature stratification. That is why we subdivided our patient population into 4 groups: group 1: females with age of onset under 35 years (age 28 ± 4 [18-35]), group 2: patients with age of disease onset above 35 years (44 ± 8 [38-60] with 1 M/6F), group 3: patients living in the reported endemic localities (32 ± 9 [18-60]), and group 4: patients originated from other southern Tunisian regions (37 ± 10 [18-58] with a sex ratio 9F/1M).

### 3.2. SNP Analysis

Genotype frequencies of all polymorphisms tested in control subjects were consistent with those expected from the HWE except for the rs3775294 polymorphism in the TLR3 gene and the microsatellite GT repeats in the TLR2 gene (*P* < 0.001).

Minor allele frequency of all polymorphisms was consistent with that reported in the Hap Map database. All genotyped SNPs were polymorphic, excluding the *TLR2* and TLR4 studied SNPs. Thus, *TLR2* exon 1 sequencing showed that all the genotyped SNPs were not polymorphic in the Tunisian population (rs743703/4/5/6/7/8 and 9), and the mutant alleles were absent in our population. Furthermore, The TLR4rs4986790 and rs4986791 showed heterozygote frequencies about 0.05 and 0.08, respectively.

The genotypic and allelic distributions of the studied SNPs as well as their association with the risk to PF are shown in Table 2.Considering the whole population, no significant difference was found in the allelic and genotypic distribution for all the studied SNPs. The patient's stratification in the four groups based on the epidemiodemographic features of the disease in our country revealed many interesting results (Table 2).

In group 1, a barely significance in the TLR3-rs574305 A allele (*P* = 0.06) was observed in patients, reaching 24.5% in female patient with age of onset under 35 years compared with 34.9% in the controls (Table 2). No statistically significant difference in the genotype and the allele distributions of the others studied SNPs were noted.

Contrary to group 1, in patients whose age of disease onset was above 35 years (group 2), a significant increase of the TLR3-rs574305 AA genotype and the A allele frequencies was observed, reaching, respectively, 21% and 39.4% compared with 6.3% and 26.8% in the controls (*P* = 0.009, OR = 3.92 [1.3-11.7] and *P* = 0.038, 1.78 [1.03-3], respectively). Regarding the TLR3-rs3775294, the allelic distribution revealed a significant decrease of the C allele in the patient group compared to HC (32.5% vs 51.9%; *P* = 0.03, OR = 0.57 [0.3-0.9]); in addition, the CC genotype showed a quiet protective effect when PF patients (16%) were compared to HC (30.7%) (*P* = 0.07). Taking in account the *TLR7* gene localization on the X chromosome, we analyzed sex association of the considered SNP and PF. In spite of the increase of the rs3853839 < GG genotype noted in women patients and the G allele in male ones compared to their relative healthy control, no statistically significant difference was revealed.

Inpatients living in endemic localities of the disease (group 3) and as for group 1, a barely significant decrease in the TLR3-rs574305A allele distribution in patients compared to HC was observed (24.3% vs 36.2%; *P* = 0.07). Also, the rs3775291 AG and the TLR7-rs3853839 GG genotypes were more frequent in patients compared to their matched HC (21% and 21.6% vs 14.9% and 16.1%, respectively) (Table 2).

On the other hand, group 4 allelic and genotypic distributions were quiet identical to group 2. Statistical analysis indicated a significant difference for the *TLR3-*rs3775294. Indeed, the rs3775294 > CC homozygous genotype was significantly more frequent in controls (23.9% vs 7.2%; *P* = 0.007, OR = 0.24, 95% CI [0.08-0.74]). Likewise, the decreased frequency of the C allele observed in the patient group (26.4%) compared to HC (45.7%) suggests its protective role against PF (*P* = 0.006, OR = 0.52, 95% CI [0.32-0.83]). In spite of absence of significance, the TLR3-rs574305 AA genotype and A allele were more frequent in patients compared to HC (19.6% and 34.8% vs 9.6% and 27%, respectively).

### 3.3. GT Repeat Analysis

Considering the whole population, fifteen different alleles composed by repeats (GT)_12_-(GT)_26_ ranging from 213 to 241 bp were revealed. In both patients and control groups, the most common alleles were (GT)_18_ (25% and 18.5%, respectively) and (GT)_19_ (23.7% and 21.4%, respectively) (Table 3). No significant differences were found in the allelic and genotypic distributions for the TLR2 (GT)_n_ microsatellite and the disease (Table 3).

The *TLR2*GT repeat distribution in the different epidemiological stratification disease groups and their respective matched control groups separately revealed that the TLR2 GT repeat seems to be closely associated with PF's risk in patients originated from the endemic localities (group 3) (Table 3). Thus, in group 3, the GT_18_ allele was significantly more prevalent in patients (34.2%) compared to HC (18.2%) (*P* = 0.02; OR = 2.3, 95% CI [1.1-4.9]). Furthermore, the GT_19_ allele was barely significantly more frequent in patients (30%) compared to HC (17%) (*P* = 0.059). To the contrary, the GT_23_ repeat could be considered as protector allele; indeed, it was significantly less frequent in patients (4.2%) compared to their matched HC (15.8%) (*P* = 0.02, OR = 0.2, 95% CI [0.06-0.87]). Besides, the heterozygous genotype GT_18_/GT_19_ seems to confer susceptibility to endemic PF. Indeed, it was the most frequent genotype in PF patient's living in endemic localities (34.2%) compared to HC (2.4%) (*P* = 2.3 10^−4^, OR = 20, 95% CI [2.5-171]). On the other hand, the division of the microsatellite alleles into two groups, according to Moore et al. [24], showed that medium GT alleles (between 18-22 repeats) that induce high promoter activity were significantly more frequent in PF patients than short or long GT repeats (≤ 17 and ≥ 23) which induce low promoter activity (*P* = 0.0018 with OR = 3.26 [1.5-7].

No statistically significant differences in the genotype and the allele frequencies concerning the *TLR 2* microsatellite were observed after patients' epidemiological stratification according to the age of onset (Table 3).

### 3.4. Linkage Disequilibrium (LD), Haplotype, and Gene Interaction

The LD analysis among the patient group was conducted by pairwise comparison of the 3 polymorphisms studied in the TLR3 gene within chromosome 4. No evidence for LD was revealed in the four groups of patients and their related matched HC. However, the rs5743305 > T, rs3775294 > C, and rs3775291 > G, which contains the rs3775294 > C protector allele, could be considered as protector haplotype to the disease in group 2 (*P* = 0.005, OR = 0.37, 95% CI [0.18-0.76]) and in group 4 (*P* = 0.039, OR = 0.58, 95% CI [0.35-0.97]), respectively. Thus, it was more expressed in controls (30.2% and 36.6%, respectively) than in patients (15.6% and 26.5%, respectively).

On the other hand, SHEsis analysis of the gene-gene interactions which were assessed for the PF patient groups and their respective controls revealed distinct results. Thus, patients from endemic localities (group 3) exhibit a barely interaction between TLR2 GT repeat and TLR3-rs5743305 (*P* = 0.081), whereas patients from sporadic regions showed some significant interactions between the GT repeat and the rs3775294 (*P* = 0.019) and the rs4986791 (*P* = 0.06).

On the other hand, female with age of disease onset was under 35 years, TLR2 GT repeat, and the TLR4-rs4986790 (*P* = 0.018).

## 4. Discussion

Considering the plethora of reports on the pattern recognition receptors, it seems plausible that TLRs might harbour susceptibility loci for the autoimmune process. A multitude of studies described the genetic variation in practically all TLRs [10]. Polymorphisms of TLRs have not been analyzed in PF disease to date. This study is, to the best of our knowledge, the first to demonstrate an association between *TLR* gene polymorphisms and PF in the Tunisian population.

Interestingly, based on the epidemiological-stratification analysis, discrepant results between endemic (groups 1 and 3) and sporadic patients' groups (groups 2 and 4) were observed which confirm that the difference in their genetic background of susceptibility to the disease can be modulated by their different epidemiological features of the disease and confirm our previous ascertainment [27, 28]. Indeed, this epidemiological-stratification analysis showed that the association between *intronic TLR2* GT repeat polymorphism and PF was pronounced in the patient's group originated from endemic localities. In sporadic patient's groups, the *TLR3*-rs5743305 confer risk to PF, whereas the *intronicTLR3*-rs3775294 was reported to have a protector role. Furthermore, our genetic analysis revealed the absence of any association between the others polymorphisms investigated in *TLR3* (rs3775291) and in *TLR7* (rs3853839) and susceptibility to PF. The ancestral allele was exclusive in all the TLR2 studied SNPs (rs743703/04/05/06/07/08 and 09). Heterozygous variants for rs4986790 (Gly299Asp) and rs4986791 (Ile399Thr) in the TLR4 gene demonstrated lower levels.

For the TLR2 (GT)_n_ microsatellite, considering the whole population, no significant differences were found in the allelic and genotypic distribution. The epidemiodemographic-stratification analysis showed that the association between *intronic TLR2* GT repeat polymorphism and PF was pronounced only in the patient's group originated from endemic localities. In fact, the GT_18_ allele and the GT_18_/GT_19_ genotype were associated with a significant risk to PF disease (*P* = 0.02 and 2.3 10^−4^, respectively), whereas the GT_23_ allele could be considered as a protector allele (*P* = 0.02). Furthermore, medium GT alleles (between 18-22 repeats) were significantly more frequent in endemic PF patients (*P* = 0.0018, OR = 3.26). Previously, Moore et al. [24] reported that higher promoter activity could be induced by medium GT alleles. So, we can speculate that the *TLR2* intronic polymorphisms could induce the regulation of the tlr2 receptor expression which might control the disease severity in the Tunisian population and make patients more prone to inflammatory diseases such as PF. This hypothesis is supported with our recent report that showed a significant increase of the tlr2 receptor expression in the PF lesional tissue compared to that of normal controls by the immunohistochemistry method [29]. Taken together, these investigations highlight the potential role of the TLR2 gene and protein in the pathogenesis of PF. tlr2 which interacts with peptidoglycan as well as additional constituents of Gram-positive bacteria, mycobacteria, and fungi [30] is shown to be immune functional receptors on KCs [31].

As to the TLR3 studied SNPs, the epidemiodemographic-stratification analysis showed that the TLR3-rs574305 AA genotype and A allele were significantly more frequent in patients whose age of the onset of disease was above 35 years (group 2) compared to their matched HC (*P* = 0.038, OR = 1.79 and *P* = 0.009, OR = 3.92, respectively). On the other hand, the *TLR3*-rs3775294 *intronic* polymorphism seems to be a protective factor in the sporadic patient's groups (groups 2 and 4). Indeed, the distributions of rs3775294 > C allele and the homozygous genotype rs3775294 > CC were significantly more prevalent in control subjects. Considering the fact that the TLR3 gene: rs5743305 (T/A) is located within the promoter region and rs3775294 (C/T) is a nonsynonymous SNP located within intron 2 which might affect the transcriptional activity [8, 9], we can hypothesize that the breakdown of this process giving variant receptor functionally impaired, and that tlr3 receptor could have a pivotal function in this pathology. The stronger diffuse expression of the tlr3 receptor throughout all layers of KCs reported in our previous data could consolidate this hypothesis [29].

For the *TLR4* mutations, notably, those situated in the fourth exon: rs4986790 and rs4986791 which alter the extracellular domain of this receptor seem to be not associated to PF in the Tunisian population. These findings do not provide explanation of previous findings that showed the significant *tlr4* expression in lesional PF patient's biopsies than in controls biopsies. Thus, the *tlr4*over expression was showed to be predominantly more pronounced in the basal layer and slightly throughout the PF's epidermis, using immunohistochemical analysis [29]. Additionally, a study in a Chinese cohort in different intraepidermal bullouse diseases showed a relocalization of tlr4 expression sites with increased expression in pemphigus and BP lesions [32]. Taken together, these findings argue for the implication of other particular polymorphisms that should enhance TLR4 signaling in PF's keratinocytes. The direct sequencing of the *TLR4* gene seems to be required to identify eventual missense mutation specific for the Tunisian population.

Finally, the present study has some limitations that should be considered. Although the *TLR 2*, *3*, *4*, and *7* polymorphisms studied were more studied polymorphism in AID, polymorphisms of TLR have not been analyzed in PF disease to date; therefore, results should be confirmed in other ethnicities, while an integrative genomic approach to infer causal associations between the gene expression and PF should be elucidated in future experiments.

*TLRs* were considered as critical genes that may shift the balance between pro- and anti-inflammatory cytokines and cause autoimmunity. The inactivation or overexpression of genes, that encode TLRs and subsequently alter TLR signaling, provide a bridge between the innate and adaptive immune systems and could constitute an important factor implicated in the development of AID.

## 5. Conclusion

In conclusion, the present study revealed a number of genetic associations between *TLR* gene polymorphisms and Tunisian PF susceptibility. The main findings emerged with *TLR2* GT repeat, TLR3-rs574305, and *TLR3*-rs3777294 which exhibit a distinct association according to the epidemiodemographic-stratification analysis, indicating their possible role in certain subphenotypes. Further, depth experimental studies and multiple center studies are needed to consolidate the correlation between PF disease and TLR gene polymorphism in the newly submitted version.

## Figures and Tables

**Table 1:** Primary information of genotyped polymorphisms in *TLR2*, *3*, *4*, and *7* genes.

Gene	SNPs	Base change	Localization	Primers	Enzyme
*TLR2*	rs5743703/04/05/06/07/09	G/A-C/A-T/C-T/A-T/G-G/A	Exon 1	F: 5′-GGCCAGCAAATTACCTGTGT-3′ R: 5′-GATCCCAACTAGACAAAGAC-3′	┼
	rs5743708	+2477 G/A (Arg753Gln)	Exon 1	F: 5′-GGCCAGCAAATTACCTGTGT-3′ R: 5′-GATCCCAACTAGACAAAGAC-3′	*Pst1*
	(GT)n	—	Intron 2	F: 5′-TATCCCCATTCATTCGTTCCATT-3′ R:5′Hex-GACCCCCAAGACCCACACC-3′	$
*TLR3*	rs5743305	-8441 T/A	Promotor	F: 5′-GGGACAGTCACGTACTCAGGA-3′ R:5′-GTGGGCTCCAGCTTCAACTA-3′	*SpeI*
	rs3775294	T/C	Intron 2	F: 5′-CACATGGCTTATCAAACACAC-3′ R:5′-CAAAGGGTGATAAAAACATCCT-3′	*Pst1*
	rs3775291	+1234 G/A (Leu412Phe)	Exon 4	F: 5′-ATCAGTCGTTGAAGGCTTGG-3′ R: 5′-TGCTCATTCTCCCTTACACAGA-3′	*Tfi1*
*TLR4*	rs4986790	+896A/G (Asp299Gly)	Exon 4	F: 5′-GATTAGCATACTTAGACTACTACCTCCATG-3′ R: 5′-GATCAACTTCTGAAAAAGCATTCCCAC-3′	*Nco I*
	rs4986791	+1196 C/T (Thr399Ile)	Exon 4	F: 5′-GGTTGCTGTTC CAAAGTGATTTTGGGACAA-3′ R: 5′-GGAAATCCAGATGTTCTAGTTGTTCTAAGCC-3′	*Hinf1*
*TLR7*	rs3853839	C/G	3′UTR	F: 5′TTGCTTCCGTGTCATCCAGG3′ R: 5′ACAGTACTTTGCAGTGCAGATAAA3′	*StyI*

┼: SNPs were genotyped by direct sequencing; $: primers as described previously [24] and was genotyped using the automatic genotyping method.

**Table 2:** Genotype and allele frequencies of *TLR3, 4* and *7* studied SNPs in Pemphigus foliaceus patient's groups and their relative matched healthy controls.

	Group 1 ♀ age of onset under 35 years			Group 2 ♀♂ age of onset above 35 years			Group 3 Endemic localities			Group 4 Nonendemic localities		
Gene/SNP	Case *N* = 55 (%)	Controls *N* = 83 (%)	*P* OR, 95% CI	Case *N* = 38 (%)	Controls *N* = 110 (%)	*P* OR, 95% CI	Case *N* = 37 (%)	Controls *N* = 69 (%)	*P* OR, 95% CI	Case *N* = 56 (%)	Controls *N* = 124 (%)	*P* OR, 95% CI
*TLR3 > rs 5743305*												
A	27 (24.5)	58 (34.9)	0.067	30 (39.4)	59 (26.8)	0.038 1.78 [1.03-3]	18 (24.3)	50 (36.2)	0.07	39 (34.8)	67 (27)	0.13
T	83 (75.4)	108 (65)		46 (60.5)	161 (73.1)		56 (75.6)	88 (63.7)		73 (65.1)	181 (72.9)	
AA	7 (12.7)	17 (20.4)	0.23	8 (21)	7 (6.3)	0.009 3.92 [1.3-11.7]	4 (10.8)	12 (17.3)	0.36	11 (19.6)	12 (9.6)	0.063
AT	13 (23.6)	24 (28.9)	0.4	14 (36.8)	45 (40.9)	0.6	10 (27)	26 (37.6)	0.26	17 (30.3)	43 (34.6)	0.5
TT	35 (63.6)	42 (50.6)	0.1	16 (42.1)	58 (52.7)	0.2	23 (62.1)	31 (44.9)	0.09	28 (50)	69 (55.6)	0.4
*TLR3 > rs3775294*												
C	36 (37)	57 (36.5)	0.9	29 (32.5)	108 (51.9)	0.03 0.57 [0.3-0.9]	31 (50)	58 (44.6)	0.4	34 (26.4)	107 (45.7)	0.006 0.52 [0.32-0.83]
T	62 (63)	99 (63.4)		47 (67.5)	100 (48)		31 (50)	72 (55.3)		78 (73.5)	127 (54.2)	
CC	7 (13)	14 (17.9)	0.5	6 (16)	32 (30.7)	0.07	9 (29)	18 (27.6)	0.8	4 (7.2)	28 (23.9)	0.007 0.24 [0.08-0.74]
CT	22 (45)	29 (37.1)	0.4	17 (45)	44 (42.3)	0.1	13 (42)	22 (33.8)	0.7	26 (46.4)	51 (43.5)	0.7
TT	20 (42)	35 (44.8)	0.6	15 (39)	28 (26.9)	0.1	9 (29)	18 (27.6)	0.8	26 (46.4)	38 (32.4)	0.07
*TLR3 > rs3775291*												
A	12 (11)	12 (7.4)	0.29	9 (12.5)	26 (12.2)	0.95	8 (10.5)	10 (7.4)	0.44	13 (12.5)	28 (11.6)	0.85
G	96 (89)	150 (92.5)		63 (87.5)	186 (87.7)		68 (89.4)	124 (92.5)		91 (87.5)	212 (88.3)	
AA	0	1 (1.2)		0	0	—	0	0	—	0	1 (0.8)	0.78
AG	12 (22.2)	10 (12.3)	0.1	9 (25)	26 (24.5)	0.9	8 (21)	10 (14.9)	0.4	13 (25)	26 (21.6)	0.63
GG	42 (77.7)	70 (86.4)	0.1	27 (74.9)	80 (75.4)	0.9	30 (78.9)	57 (85)	0.4	39 (74.9)	93 (77.5)	0.7
*TLR4 > rs4986790*												
A	98 (94.2)	148 (91.3)	0.38	73 (96)	201 (95.7)	0.89	70 (94.5)	124 (92.5)	0.57	101 (95.2)	225 (94.5)	0.77
G	6 (5.7)	14 (8.6)		3 (3.9)	9 (4.2)		4 (5.4)	10 (7.4)		5 (4.7)	13 (5.4)	
AA	46 (88.4)	68 (83.9)	0.6	35 (92.1)	97 (92.3)	0.79	33 (89.1)	58 (86.5)	0.69	48 (90.5)	107 (89.9)	0.9
AG	6 (11.5)	12 (14.8)		3 (7.8)	7 (6.6)		4 (10.8)	8 (11.9)	0.86	5 (9.4)	11 (9.2)	0.9
GG	0	1 (1.2)		0	1 (0.9)		0	1 (1.4)	0.45	0	1 (0.8)	0.13
*TLR4 > rs4986791*												
C	99 (95.1)	109 (95.6)	0.8	74 (97.3)	190 (97.9)	0.77	71 (95.9)	85 (94.4)	0.72	102 (96.2)	214 (98.1)	0.44
T	5 (4.8)	5 (4.3)		2 (2.6)	4 (2)		3 (4.1)	5 (5.6)		4 (3.7)	4 (1.8)	
CC	47 (90.3)	52 (91.2)	0.87	36 (94.7)	93 (95.8)	0.67	34 (91.8)	40 (88.8)	0.65	49 (92.4)	105 (96.3)	0.28
CT	5 (9.6)	5 (8.7)	0.87	2 (5.2)	4 (4.1)	0.67	3 (8.1)	5 (11.1)	0.65	4 (7.5)	4 (3.6)	0.28
TT	0	0	—	0	0	—	0	0	—	0	0	—
*TLR7 > rs3853839*												
Female												
C	57 (53.7)	85 (57.3)	0.56	45 (62.4)	124 (64.2)	0.79	42 (56.7)	71 (57.2)	0.94	60 (57.6)	138 (63.5)	0.28
G	49 (46.2)	62 (41.8)		27 (37.4)	69 (35.7)		32 (43.2)	53 (42.7)		44 (42.3)	78 (35.9)	
CC	18 (33.9)	22 (29.7)	0.33	13 (36.1)	33 (34.1)	0.81	13 (35.1)	19 (30.6)	0.64	18 (34.6)	36 (33.1)	0.9
CG	21 (39.6)	38 (51.3)	0.1	16 (44.4)	47 (48.7)	0.67	16 (43.2)	33 (53.2)	0.33	21 (40.3)	52 (47.9)	0.26
GG	14 (26.4)	12 (16.2)	0.18	4 (11.1)	9 (9.3)	0.7	8 (21.6)	10 (16.1)	0.78	10 (19.2)	11 (10.1)	0.12
Male												
C	—	—		3 (50)	14 (67)	0.4				3 (50)	14 (67)	0.4
G	—	—		3 (50)	7 (33)					3 (50)	7 (33)	

This case control-study enrolled 93 PF patients matched to 193 healthy control, whereas in some subjects the genotyping was failed.

**Table 3:** Genotype and allele frequencies of *TLR2 GT repeat* inPemphigus foliaceus patient's groups and their relative matched healthy controls.

	Group 1 ♀ age of onset under 35 years			Group 2 ♀♂ age of onset above 35 years			Group 3 Endemic localities			Group 4 Nonendemic localities		
	Case *N* = 55 (%)	Controls *N* = 83 (%)	*P* OR, 95% CI	Case *N* = 38 (%)	Controls *N* = 110 (%)	*P* OR, 95% CI	Case *N* = 37 (%)	Controls *N* = 69 (%)	*P* OR, 95% CI	Case *N* = 56 (%)	Controls *N* = 124 (%)	*P* OR, 95%CI
*TLR2>(GT)n*												
12	2 (1.9)	4 (4.9)	0.2	2 (3.4)	3 (1.8)	0.5	1 (1.4)	5 (6)	0.1	3 (3.2)	1 (0.6)	0.1
13	7 (6.7)	4 (4.9)	0.6	5 (8.6)	5 (3.1)	0.09	6 (8.5)	3 (3.6)	0.2	6 (6.5)	6 (3.8)	0.3
14	0	1 (1.2)	—	0	1 (0.6)	—	0	1 (1.2)	—	0	1 (0.6)	0.4
15	0	0	—	0	1 (0.6)	—	0	0	—	0	1 (0.6)	0.4
16	1 (0.9)	1 (1.2)	0.8	0	5 (3.1)	—	1 (1.4)	4 (4.8)	0.2	0	2 (1.2)	0.27
17	3 (2.8)	4 (4.9)	0.45	5 (8.6)	10 (6.3)	0.5	1 (1.4)	5 (6)	0.1	7 (7.6)	9 (5.8)	0.58
18	28 (26.9)	16 (19.9)	0.27	13 (22.4)	28 (17.7)	0.4	24 (34.2)	15 (18.2)	0.02 2.3 [1.1-4.9]	17 (18.4)	29 (18.8)	0.9
19	25 (24)	21 (26..2)	0.7	14 (24.1)	30 (18.9)	0.4	21 (30)	14 (17)	0.059	18 (19.5)	37 (24)	0.5
20	12 (11.5)	10 (12.4)	0.8	7 (12)	16 (10.5)	0.6	8 (11.4)	5 (6)	0.2	11 (11.9)	21 (13.6)	0.7
21	8 (7.6)	3 (3.7)	0.26	1 (1.7)	17 (10.7)	0.03	4 (5.7)	12 (14.6)	0.07	5 (5.4)	8 (5.1)	0.9
22	4 (3.8)	4 (4.9)	0.7	2 (3.4)	9 (5.6)	0.5	1 (1.4)	3 (3.6)	0.4	5 (5.4)	10 (6.4)	0.7
23	11 (10.5)	11 (13.7)	0.5	6 (10.3)	22 (13.9)	0.48	3 (4.2)	13 (15.8)	0.02 0.2 [0.06-0.87]	14 (15.2)	19 (12.3)	0.5
24	1 (0.9)	0	—	2 (3.4)	4 (2.5)	0.7	0	1 (1.2)	—	3 (3.2)	3 (1.9)	0.5
25	1 (0.9)	1 (1.2)	0.8	1 (1.7)	5 (3.1)	0.56	0	1 (1.2)	—	2 (2.1)	5 (3.2)	0.6
26	1 (0.9)	0	—	0	2 (1.2)	0.38	0	0	—	1 (1)	2 (1.2)	0.7
GT18/GT19	12 (23)	2 (4.9)	0.16	6 (20.6)	6 (7.5)	0.054	12 (34.2)	1 (2.4)	2.3 10^−4^ 20 [2.5-171]	6 (13)	7 (11)	0.5
GT18/GT20	5 (9.6)	0	0.043	3 (10.3)	2 (2.5)	0.08				5 (10.8)	1 (1.2)	0.017
18 ≤ Gn ≤ 22	77	54	0.47	37	100	0.9	58	49	0.0018 3.26 [1.5-7]	56	105	0.052
GTn ≤ 17 GTn ≥ 23	27	25		21	58		12	33		36	49	

This case control-study enrolled 93 PF patients matched to 193 healthy control, whereas in some subjects the genotyping was failed.

## Data Availability

The datasets generated and analyzed for the current study are available at the affiliated Hospital (Habib Bourguiba University Hospital of Sfax). The datasets used and analyzed during the current study are available from the corresponding author on reasonable request.

## Abbreviations

PF: Pemphigus foliaceus
TLR: Toll-like receptors
Abs: Antibodies
Dsg1: Desmoglein-1
HC: Healthy controls
SNPs: Single nucleotides polymorphisms
HWE: Hardy–Weinberg equilibrium
OR: Odds ratios
CI: Confidence intervals
LD: Linkage disequilibrium.
